# Sudden Sensorineural Hearing Loss Following Electric Shock: A Case Report with Literature Review

**DOI:** 10.22038/ijorl.2024.77019.3579

**Published:** 2025

**Authors:** Arshit Kataria

**Affiliations:** *Senior Resident, Department of ENT and * *Head & Neck Surgery* *, All India Institute of Medical Sciences, Bathinda, Punjab, India.*

**Keywords:** Case report, Electric shock, High-voltage, Pure tone average, Sensorineural

## Abstract

**Introduction:**

Electric shock occurs when electricity passes through the body, causing a range of symptoms from mild tingling to potentially life-threatening injuries such as burns, seizures, and cardiac arrest. In rare cases, Sudden Sensorineural Hearing Loss (SSNHL) has also been associated with an electric shock.

**Case Report:**

A 35-year-old male presented with left-sided hearing loss following an electric shock. The audiometric evaluation revealed left Sensorineural hearing loss (SNHL). The patient was prescribed oral corticosteroids showing some improvement in hearing thresholds and was advised hearing aid for the residual SNHL.

**Conclusion:**

Limited literature exists on the association between high-voltage electric shocks and hearing loss. This case report highlights the importance of prompt evaluation and management of (SSNHL) in individuals affected by electric shock.

## Introduction

Electric shock is a sudden and often intense physiological response to an electrical current passing through the body. It can occur when a person comes into contact with an electrical source, such as an exposed wire or an appliance with a faulty electrical connection. The severity of an electric shock can range from a mild tingling sensation to a potentially life-threatening injury, depending on the voltage, current, duration, and pathway of the electrical current through the body. Symptoms of electric shock can include burns, muscle contractions, seizures, loss of consciousness, and cardiac arrest. Every year, numerous individuals experience electrical injuries, with several thousands of people affected. In the United States, approximately, 1000 people pass away annually as a result of lightning strikes and other types of electrical injuries (1). In rare cases, SSNHL has also been associated with an electric shock. We have described the case of a 35-year-old male who acquired left-sided SSNHL following an electric shock. This case report was prepared following the CARE guidelines.

This study was conducted following the ethical standards set forth by our Institutional Review Board (IRB). The IRB approved the study and provided oversight throughout the study to ensure that all procedures complied with established ethical guidelines.

## Case Report

A 35-year-old male presented to the emergency department in our hospital with burns on his right hand following an electric shock while installing a tube light in the house. The patient was conscious and oriented with fair general conditions and vital signs. The patient was admitted to the Department of Burns and Plastic Surgery for observation. The next day, while assessing the patient for discharge, he complained about left-sided decreased hearing with aural fullness for which ENT consultation was done.

**Fig 1:**
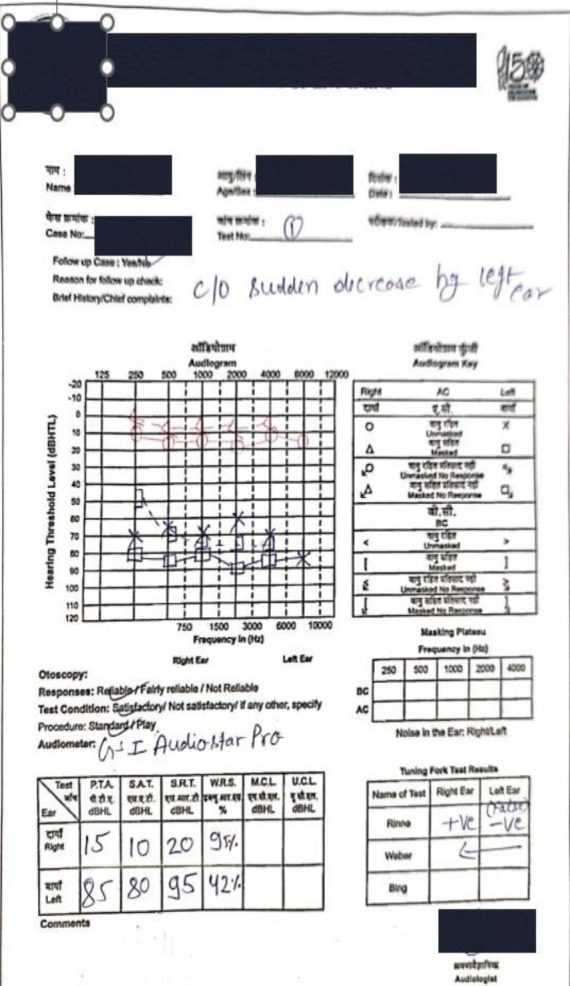
Pure tone audiometry findings of the patient at the initial presentation

The patient was assessed by the ENT department. A detailed history was taken. There was no history of hard of hearing before the electric shock incident. No history of tinnitus or vertigo. No history of drug intake other than intravenous fluids during admission to the plastic surgery department was there. The examination of the ear and other evaluations related to the ear, nose, and throat appeared to be without any abnormalities. The patient underwent an audiometric evaluation in our department. The results of the audiometry revealed left SNHL with a pure tone average of 15 dB and 85 dB for the right and left ear respectively (Figure 1).

**Fig 2:**
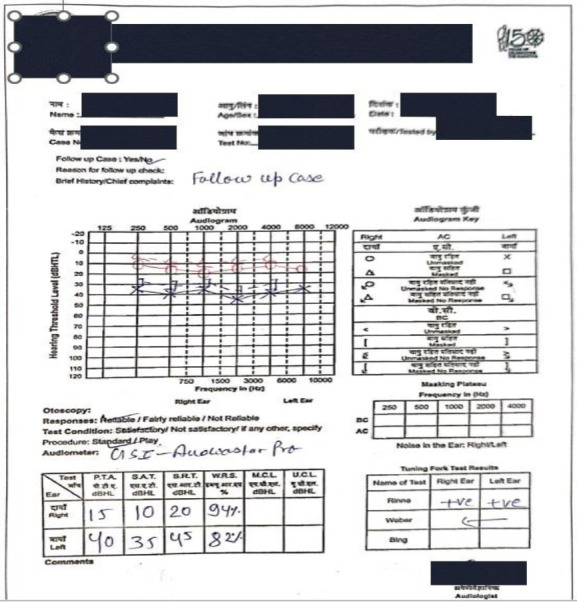
Pure tone audiometry findings of the patient after steroid therapy

Speech Discrimination Score (SDS) was 95% in the right ear and 42% in the left ear, while their Speech Reception Threshold (SRT) was measured at 20 dB and 95 dB for the right and left ears, respectively. Tympanometry indicated a Type A curve, which signifies normal eardrum function. High-resolution computer tomography and contrast-enhanced Magnetic resonance imaging were done which came out to be normal. The patient was prescribed oral prednisolone 1mg/kg/day for 14 days which was then tapered. The Pure tone averages of the left ear on the 15th day were improved to 40 dB (Figure 2). 

The SDS and SRT of the left ear were 82% and 45 dB respectively. The patient was advised with conventional hearing aids for the left ear. A follow-up was conducted after 1, 6, and 18 months, and the audiometric results were found to be similar to those observed on the 15th day.

## Discussion

The term "Sudden sensorineural hearing loss" (SSNHL) is typically used to describe a type of hearing loss that occurs suddenly and affects the inner ear. Specifically, it is characterized by a reduction in hearing sensitivity of at least 30 decibels (dB) across three or more adjacent frequencies on a hearing test, and this change in the hearing must happen within 72 hours. Otologic injury from lightning has been sometimes notified, with perforation of the tympanic membrane (more than 50%) being the most common injury, along with ossicular disruption, vestibular organ injury with transient vertigo, tinnitus, and sensorineural hearing loss. The intensity of the lightning strike fluctuates based on factors such as the duration of the shock, the anatomical points of contact, and the pathways through which the current flows (2). How damage occurs to the auditory system is uncertain and may differ in each instance. There are various potential ways in which damage can occur, such as the tearing of the eardrum due to loud noises, the passage of electric current through the cochlea, alterations in blood flow, and bleeding (3).

B Barber et al. has concluded that sensorineural deafness occurs when the acoustic shock wave passes through the tympanic membrane and middle ear, resulting in trauma to the inner ear (4). The disruption of the inner ear's anatomy, along with microhemorrhage and microfractures within the cochlea, could potentially be accountable (5). The cochlea is unlikely to receive an electric current from the external auditory meatus and middle ear due to the bone enclosure. Instead, the electric current may pass through the internal auditory meatus and cerebrospinal fluid.3 Nerve damage is a direct consequence of electrical shock injury. Peripheral mononeuropathies or polyneuropathies are common sequelae of electrical injury (6).

The available literature on the relationship between high-voltage electric shocks and hearing impairment is limited and the audio vestibular consequences following electrical injuries frequently occur unexpectedly and are likely far more prevalent than reported in the literature (7).

## Conclusion

To our knowledge, this is one of the few reported cases of SSNHL associated with an electric shock. Through this case report, we aim to increase awareness of this rare but potentially devastating complication of electric shock and emphasize the importance of prompt evaluation and management of SSNHL in affected patients.

## Patient’s Perspective

I was installing a tube light in my house when I experienced an electric shock. I was immediately brought to the hospital where I was admitted. The next day I was unable to hear properly from my left ear for which I was shown to the ear specialist. My ear was checked and I underwent some tests related to my hearing. I got worried after knowing that investigations are in favor of sensorineural hearing loss. I was given oral drug therapy for some days after which I was called back by the doctor to test again for my hearing. Hearing tests showed some improvement. My doctor explained my condition and advised me hearing aid for my left ear and advised me for a regular follow-up. I am thankful to my doctor for guiding me.
